# Diatom Valve Three-Dimensional Representation: A New Imaging Method Based on Combined Microscopies

**DOI:** 10.3390/ijms17101645

**Published:** 2016-09-28

**Authors:** Maria Antonietta Ferrara, Edoardo De Tommasi, Giuseppe Coppola, Luca De Stefano, Ilaria Rea, Principia Dardano

**Affiliations:** Institute for Microelectronics and Microsystems, Department of Naples, National Research Council, Naples 80131, Italy; edoardo.detommasi@na.imm.cnr.it (E.D.T.); giuseppe.coppola@cnr.it (G.C.); luca.destefano@cnr.it (L.D.S.); ilaria.rea@na.imm.cnr.it (I.R.)

**Keywords:** diatoms, three-dimensional image processing, digital holography, scanning microscopy

## Abstract

The frustule of diatoms, unicellular microalgae, shows very interesting photonic features, generally related to its complicated and quasi-periodic micro- and nano-structure. In order to simulate light propagation inside and through this natural structure, it is important to develop three-dimensional (3D) models for synthetic replica with high spatial resolution. In this paper, we present a new method that generates images of microscopic diatoms with high definition, by merging scanning electron microscopy and digital holography microscopy or atomic force microscopy data. Starting from two digital images, both acquired separately with standard characterization procedures, a high spatial resolution (Δ*z* = λ/20, Δ*x* = Δ*y* ≅ 100 nm, at least) 3D model of the object has been generated. Then, the two sets of data have been processed by matrix formalism, using an original mathematical algorithm implemented on a commercially available software. The developed methodology could be also of broad interest in the design and fabrication of micro-opto-electro-mechanical systems.

## 1. Introduction

Diatoms represent most phytoplankton and are responsible for about 25% of global primary production. Their chloroplast is enclosed in a porous silica shell, the frustule, made by two valves interconnected by a lateral girdle [1]. More than 10^5^ species have been identified so far, differing in dimensions (some microns up to 1 mm), shapes, pore diameters (from some nanometers to some microns) and pore distributions. In the 19th century, diatom frustules represented the standard objects to test the quality of microscope optics. Moreover, the frustule of some diatoms has showed very interesting photonic properties, generally related to its complicated and quasi-symmetric micro- and nano-structure, behaving as a natural photonic crystal [1,2,3]. In order to simulate light propagation inside these natural structures, to study the mechanical properties and to generate three-dimensional (3D) models for synthetic replica, it is important to determine their real 3D image with a high resolution. In this way, it is possible to model the natural microsystems in biomimicry or just for the understanding of physical phenomena occurring in biological organisms.

Generally, these models are retrieved by making use of cumbersome numerical simulations or starting from high-resolution microscope images.

This step necessarily precedes the execution of computer simulations of microsystem performances, carried out to optimize the size and all the geometrical parameters of the object.

Traditional imaging methods exploit microscopes or complex optical systems, such as interferometers. In the case of images produced by optical microscopes, the resolution is affected by the diffraction limit which can reach, with the proper choice of wavelength and objectives, about 200 nm. In addition, the depth of field is de facto useless for the reconstruction of a 3D model. The resolution of scanning electron microscopy (SEM) depends on the electromagnetic properties of the sample and on the electron beam conditions, obtaining images with a resolution of a few nanometers (~10^−9^ m). In this case, the depth of field (*D*) in SEM images is *D* (mm) ≈ 0.2/α*M*, where α is the beam divergence, and *M* is the magnification. However, numerical three-dimensional information cannot be simply extrapolated by the SEM image: some algorithms reconstructing a 3D model of an object from SEM images have been recently presented in References [4,5,6,7,8]. In these works, the authors obtained a resolved and faithful 3D representation of the object by increasing the angular frequency and the angular interval (e.g., from −90° to 90°) of the acquired images. The main drawback is that these techniques require a calibration on a reference sample characterized with a different technique, such as atomic force microscopy (AFM). Moreover, the SEM microscope needs an eucentric goniometer with high definition in order to maintain the same field of view in all tilted images. Furthermore, when soft biological samples have to be imaged, a cooling stage is needed to immobilize them.

To obtain high resolution with a good field of view, combinations of two different techniques have been proposed in References [9,10,11]. In References [9,10] the 3D diatom models have been reconstructed after thin cuts or abrasion by focused ion beam (FIB) and by collecting a SEM image of each section, respectively. In Reference [11], the SEM images are combined by commercial software with a volumetric model obtained with 123 acquisitions of the confocal laser scanning microscope (CLSM). Such techniques are destructive when abrasion is involved or, in the case of the presence of fluorescent markers, imply an undesired chemical modification of the sample.

In this work, a 3D representation of single valves coming from different diatom species has been obtained through a new method [12]. This approach is based on the fusion of high planar resolution microscopy techniques, such as SEM, with high axial resolution microscopy techniques, such as digital holographic microscopy (DHM) or AFM. This new technique thus enables the modeling and the visualization of microscopic 3D objects (provided with nanoscopic details) with high resolution in all spatial directions [12].

## 2. Results and Discussion

In this paper, a 3D representation of the valves of some diatom species obtained through a new method allowing the modeling and the visualization of microscopic 3D objects with high resolution was studied [12], and results obtained on *Arachnoidiscus*, *Coscinodiscus wailesii*, and *Cocconeis* genus valves are reported. Our original method used a combination of SEM with DHM, first, and as extension of the method, a combination of SEM with AFM [12]. The method was non-destructive and label-free. Moreover, the procedure, at least for the combination of SEM and holographic imaging, was not time-consuming with respect to other methods that require a z-scan to obtain a 3D image: both SEM and DHM images were obtained in separate single acquisitions; in image processing, data were easily manipulated as matrices with the same order of magnitude of image pixels. The combination of DHM and SEM was able to produce 3D models for accurate design and numerical simulations. The same procedure can be applied to the AFM imaging in combination with SEM. In this case, the higher acquisition time required by the AFM imaging is compensated by the higher resolution in the *xy* plane.

### 2.1. Example 1: Creating a 3D Model of an Arachnoidiscus Diatom Valve

In this example, the micrometric object to reconstruct was the inner plate of a single valve from the *Arachnoidiscus* diatom, which consists of amorphous hydrated nanoporous silica and is characterized by an almost perfect circular shape [13,14]. The frustules of the *Arachnoidiscus* genus are heterovalvar, i.e., they are provided with two different valves, and the whole frustule is petri dish–shaped with an average diameter of about 200 µm. The external part of the valve is characterized by a planar central area and the inner one by a center ringed flange with elongated radial slits. In both sides of the valve an ultrastructure was present, with progressively reduced micrometric and sub-micrometric porous features.

**Figure 1:**
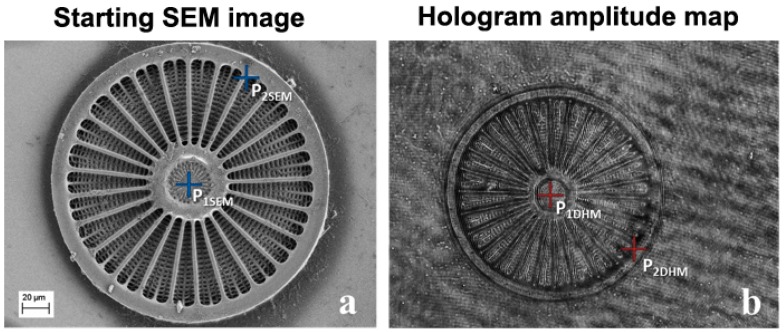
(**a**) SEM image; (**b**) Amplitude map obtained by means of DHM of an *Arachnodiscous* single valve. The P_1SEM_, P_2SEM_ and P_1DHM_, P_2DHM_ points (see main text for definition), useful for image alignment, are shown by blue and red crosses, respectively.

Figure 1 shows both the SEM image of the valve (Figure 1a) and the amplitude map, obtained by means of DHM, of the same valve (Figure 1b). The difference in resolution was noticeable. The four selected points needed to perfectly match the two images of the valve (two points in the amplitude map retrieved by DHM and two corresponding points in the SEM image) are also shown. In both images the central point and a point on the inner crown of the valve were selected.

**Figure 2:**
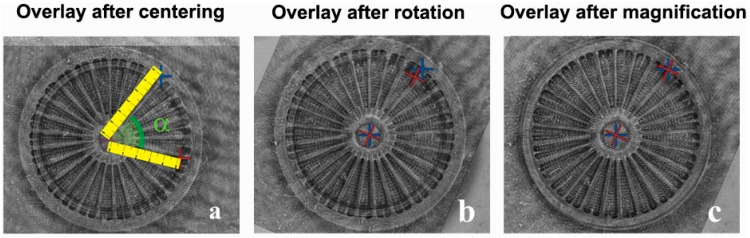
(**a**) Transparent overlay of the SEM and DHM images after their centering. The pictures underline the different orientation (given by the angle α and sizing of the same valve in the two images; (**b**)Transparent overlay of the images after rotation; (**c**) magnification adjustements.

As described in the Methods section, by applying the mathematical procedure to one of the two matrices, it was possible to translate, rotate and magnify the valve in one image with respect the other one (Figure 2).

Translation, rotation and zoom steps of the original algorithm are shown in Figure 2a–c, respectively.

**Figure 3:**
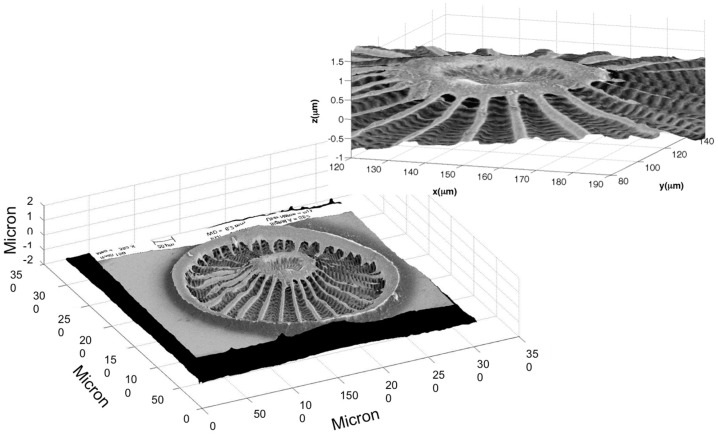
Final reconstruction by the SEM and DHM merging of the *Arachnodiscus* valve. In the inset, a zoomed view of the center of the diatom reconstruction is shown.

Finally, the texture of the 3D reconstruction of the phase map of the valve, retrieved by means of DHM, with the corresponding SEM image was obtained. In Figure 3 the three-dimensional reconstruction of the valve after proper translation, rotation and magnification operations is shown.

The final result was a full 3D view of the sample, useful for imaging and modeling purposes. In previous works [3,15,16], the proper extruding of a SEM image of the valve allowed the researchers to obtain only an approximate 3D model, useful just for rough simulations of light propagation. On the other hand, our method allows us to obtain a real, full 3D model, able to completely describe the non-uniform morphology of the valve. Such a model could be implemented in numerical simulations for light propagation in complex micro- and nano-structured media.

### 2.2. Example 2: Creating a 3D Model of a Cocconeis Diatom Valve

In this other example, a *Cocconeis* diatom valve consisting of an almost oval shape in silica was examined.

Diatoms of the genus *Cocconeis* belong to the Pennates order, characterized by a bilateral symmetry of the valves [13,14]. They are heterovalvar, one valve (*R*-valve) with a raphe sternum, the other (*P*-valve) lacking a raphe but with a corresponding rapheless sternum (“pseudoraphe”). *Cocconeis* diatoms possess one unique plastid, which is flat and C-shaped [13,14]. Valves are elliptical or almost circular. The *R*-valve is usually less convex than the *P*-valve. Similarly to what was done for the *Arachnoidiscus* diatom, we acquired a SEM image and a holographic image (DHM) of a *Cocconeis* single valve. By using the previously described mathematical approach applied to the holographic image, we reconstructed both the amplitude and the phase map, which allowed the creation of a three-dimensional model of the diatom.

**Figure 4:**
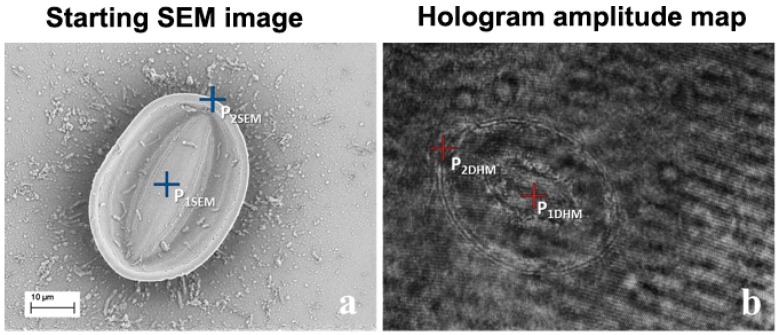
(**a**) SEM image; (**b**) Amplitude map obtained by means of DHM of the *Cocconeis* diatom. The P_1SEM_, P_2SEM_ and P_1DHM_, P_2DHM_ points (see main text for definition), useful for image alignment, are shown by blue and red crosses, respectively.

In this example, the central point and a vertex on the edge of the oval diatom in both images were selected (see Figure 4).

In order to achieve perfect alignment of the diatom in both images, the mathematical procedure described above was applied to translate, rotate and magnify the valve in one of the two images.

**Figure 5:**
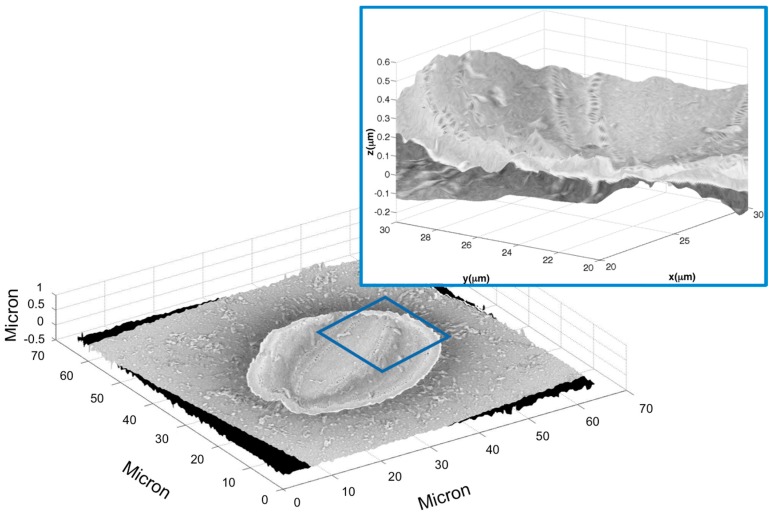
Final reconstruction by the SEM and DHM merging of the *Cocconeis* diatom. In the inset, a zoomed view of the region of the diatom reconstruction highlighted is shown.

Finally, the overlap of the SEM image of the valve on a three-dimensional reconstruction of the same valve, obtained by the holographic technique (major axis ≈ 50 μm, thickness ≈ 0.5 μm), was realized, as shown in Figure 5.

### 2.3. Example 3: Creating a 3D Image of a Porous of Coscinodiscus wailesii Diatom

The same procedure was used in the case of transverse dimension measurements performed with alternative techniques. In fact, through our procedure, it was possible to “spread” the SEM image (rotated, magnified and translated) on the measurement of transverse size obtained by AFM. In this way, our method will also be extended to objects of submicron dimensions. AFM needs longer acquisition times and represents a vantage point with respect to DHM only when the retrieval of local topography is required. In fact, the AFM is completely useless to characterize an object on the micrometric scale, such as a diatom valve, because the resolution strongly decreases as the field of view increases, because the number of pixels is fixed. Conversely, decreasing the field of view on a few pores, the resolution is very high.

*Coscinodiscus wailesii* is a typical centric diatom characterized by radial symmetry of the frustule and its pore patterns [13,14]. The whole frustule, made of hydrogenated porous silica and formed by two circular valves linked by a lateral girdle, has a quasi-cylindrical shape*. C. wailesii* valves are made of two overlapping layers: the external one is characterized by a complex array of pores about 250 nm in diameter and a lattice constant of about 500 nm, while the internal layer exhibits pores with a diameter of 1.2–1.5 µm distributed with hexagonal symmetry with a lattice constant of about 2 µm [13,14].

**Figure 6:**
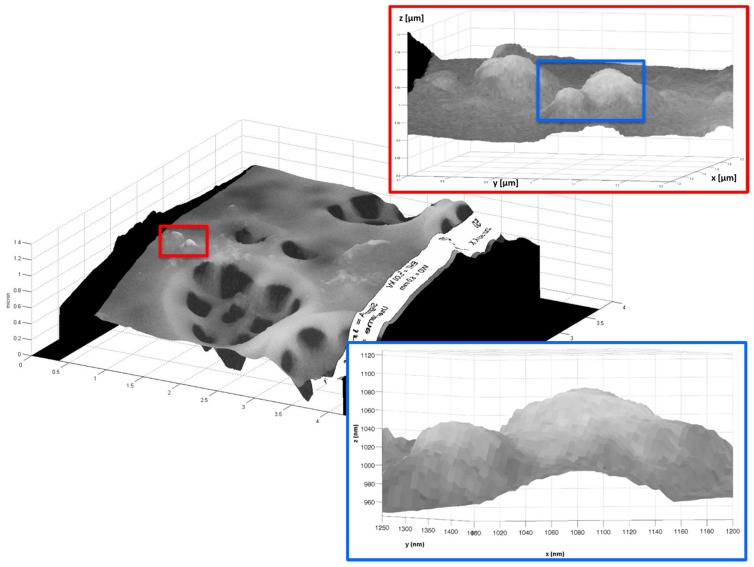
Final reconstruction by AFM and SEM merging of single pores of a *Coscinodiscus wailesii* valve. In the insets, zoomed views of a particular part of the reconstruction are shown.

In Figure 6, the merger between AFM and SEM images of a portion of a *Coscinodiscus wailesii* valve is presented. In particular, in the insets it is clear that nanometric objects are well reconstructed with this technique.

However, it should be noticed that in 3D measurement tools, such as AFM, the maximum resolution is typically tied to a limitation in the field of view. Therefore, it is impossible to capture a large object with high detail definition with this single technique. Moreover, AFM is more time-consuming as the resolution is higher.

Conversely, our method is applicable to images obtained with any microscopy technique, such as fluorescence microscopy; in this case, the image can be merged with the DHM measurement, losing definition but achieving the additional information of the fluorescence map.

## 3. Materials and Methods

### 3.1. Sample Origin and Preparation

Single valves from *Arachnoidiscus* and *Cocconeis* genera have been obtained from the AM671 sample of the Hustedt collection [13]. Coming from a historic collection, these valves did not need any removal of organic material before suspending them in a buffer solution. *Coscinodiscus wailesii* valves indeed needed another treatment. Inocula of this species have been purchased from the Culture Collection of Algae and Protozoa of the Scottish Association for Marine Science, Oban, UK.

Diatoms were grown in Guillard “f/2” medium with addition of silica at a temperature of 18 °C. Illumination consisted in 14:10 light-dark (LD) cycle with an irradiance of 30 ÷ 50 micro-mol photons m^−2^·s^−1^. Organic content was then removed from the cells according to von Stoch’s method [17], which allowed also the separation of the various frustule components.

The obtained, cleaned, sparse valves, characterized by an average diameter of 210 microns for *Arachnoidiscus* genus, 190 microns for *C. wailesii* species, and an average length of 40 microns for *Cocconeis* genus, were firstly immersed in a buffer solution, then deposited by drop casting on a tin-doped indium oxide (ITO) coated quartz glass, that ensured both the transparency, needed for DHM characterizations in transmission, and the conductivity, needed for SEM imaging.

### 3.2. Digital Holography Microscopy

**Figure 7:**
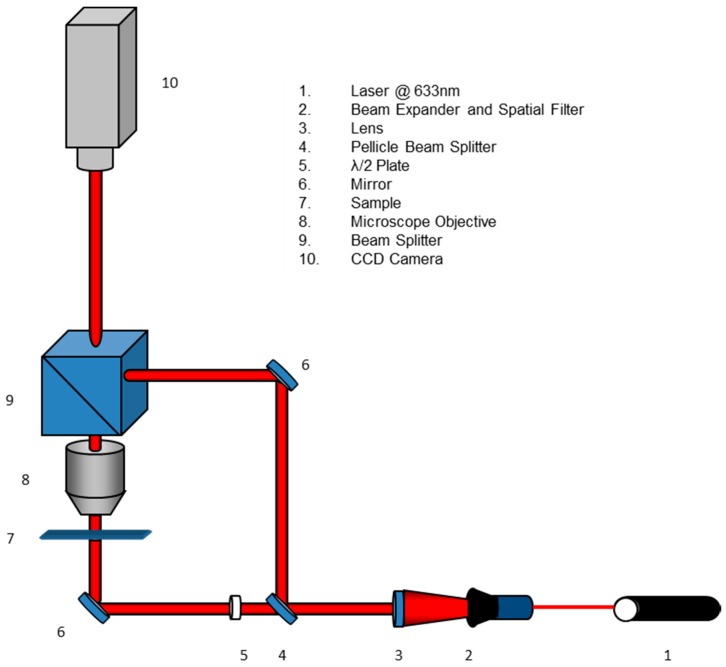
Experimental set-up for digital holographic characterization.

The DHM is able to reconstruct objects directly in 3D. Using a laser source with high spatial and temporal coherence in an interferometric configuration, special images, called holograms, with in-plane resolution of the order of microns, are produced. Currently, the DHM is used not only for morphology characterization of many micrometric objects, such as marine diatoms, or cells, but also to reconstruct the light propagation in transparent object [2,3,18,19]. In Figure 7 is reported the sketch of the experimental set-up used for the digital holographic characterization. The laser source was a Helium-Neon laser (λ = 632.8 nm), with an output power of 30 mW in continuous wave (CW). The reference and object beams, obtained by splitting the laser source with a pellicle beamsplitter, were plane wavefronts achieved by a beam expander. A λ/2 wave plate was in the object beam to obtain equal polarization direction for the two beams and, thus, to improve the fringe contrast. The object beam was collected by using a microscope objective with a magnification and numerical aperture (NA) of 10× and NA = 0.25, respectively. The two beams were recombined by a second beamsplitter and the resulting interference pattern has been collected onto the surface of a CCD camera (1392 × 1040 pixels array; each pixel had dimension Δ*x* = Δ*y* = 4.7 µm). The resolution of the 3D model of DHM is related to the microscopy resolution in the xy plane, whereas along z direction (i.e., the light propagation direction) is related to the wavelength (Δ*z* = λ/20).

### 3.3. Scanning Electron Microscopy

SEM images were performed at 5 kV accelerating voltage and 30-µm-wide aperture by a Field Emission Scanning Electron Microscope (Carl Zeiss NTS GmbH 1500 Raith FESEM, Carl Zeiss, Oberkochen, Germany). Secondary emission detector has been used.

### 3.4. Atomic Force Microscopy

AFM measurements have been performed by means of XE-70 microscope from Park Systems. The instrument is provided with two independent, closed-loop XY and Z flexure scanners for sample and tip, respectively. Flat and linear XY scan of up to 100 × 100 μm with low residual bow is provided. Out of plane motion is less than 2 nm over entire scan range. Z-scan is up to 25 μm by high force scanner. For more details about the materials, in particular for the *Arachnoidiscus* frustule and the DHM, SEM and AFM methods, we refer to our previous paper recently published [3].

### 3.5. Mathematical Procedure

The 3D images and models of the diatom valves were obtained by merging a SEM image and a digital hologram, the last being generally acquired with different orientation and magnification. The new mathematical procedure [12] was implemented by commercial software, such as Matlab^®^ or Mathematica^®^.

Starting from the acquisition of a holographic image (DHM), amplitude and phase maps of the acquired hologram have been reconstructed by means of a mathematical procedure described in Reference [12]. The phase map allowed us to obtain a three-dimensional view of the diatom.

The SEM and the DHM images have been generated by means of different sensors, thus the two images (and consequently the DHM 3D model) had a different number of pixels. Each image has been associated to a numerical matrix, M for the SEM and N for the DHM images, respectively. The dimensions of M and N are given by the pixel size of the images, while the values of their elements correspond to a gray scale (1–255) for SEM images, and values of the reconstructed 3D phase map for the DHM image. With the aim to match the size of the matrices, some constant values had to be added to the matrix of lower dimension and, as a consequence, some compensating pixels appear in the smaller image. These additional pixels had no effect on the final result because they mostly did not belong to the object, but to the background. Furthermore, they result very useful in computation, since allow a quick handling of massive quantities of data. To this purpose, fixed constants (corresponding to black or white pixels) or values calculated for continuity of the outer edge can be added to the smaller matrix.

In order to properly align the two images, we selected four points: two points in the amplitude map obtained by means of DHM, P_1DHM_ and P_2DHM_, and the corresponding two points in the SEM image, P_1SEM_ and P_2SEM_. The error in identifying the same points is related to the smallest in-plane resolution of the DHM. However, the in-plane resolution of the merging is improved by the SEM image and the axial resolution, given by the DHM reconstruction, is not affected by the choice of the points.

The P_1DHM_, P_2DHM_, P_1SEM_ and P_2SEM_ points correspond to the a_i1M,j1M_, b_i2M,j2M_, elements in M matrix and a_i1N,j1N_, b_i2N,j2N_ in N matrix.

In the following procedure, the point P_1_ in both images is usually placed on the center of the object and the points P_2_ on the edge. Applying a mathematical procedure to one of the two matrices, it is possible to translate, rotate and magnify the object in one image with respect to the other one. In the following, the above-mentioned mathematical procedure is applied to the M matrix, without loss of generality. The final result is a new matrix, M”, created from M, so that the two selected points will have the same matrix indices both in the new M” matrix and in the other matrix N:

*i*_1M’’_ = *i*_1N_, *i*_2M’’_ = *i*_2N_ (1)

*j*_1M’’_ = *j*_1N_, *j*_2M’’_ = *j*_2N_ (2)

Consequently, all the points have been consistently processed. In the following, all the over mentioned operations are described:

(a) Translation: the deviations between the indices of the P_1_ points of the two matrices Δ*i* and Δ*j* are calculated as follows:

Δ*i* = *i*_1M_ − *i*_1N_ (3)

Δ*j* = *j*_1M_ − *j*_1N_ (4)

Then, a new matrix is built as follow:

M’(*i*, *j*) = M(*i* + Δ*i*, *j* + Δ*j*) (5)

(b) Rotation: let α be the angle between the segments $\bar{P_{1\mathrm{DHM}}P_{2\mathrm{DHM}}}$ and $\bar{P_{1\mathrm{SEM}}P_{2\mathrm{SEM}}}$, in order to overlap two images/matrices, one of them is rotated around its central point P_1_ by α with respect to the other. The angle α is calculated as:

*A* = tan^−1^(*i*_2M_/*j*_2M_) − tan^−1^(*i*_2N_/*j*_2N_) (6)

The new image M’ is enlarged enough to contain the entire rotated image and the values of pixels in M’ are calculated using the nearest neighbor interpolation and setting to zero that are outside the rotated image.

(c) Zoom: the distances between the two selected points in each image, i.e., $\bar{P_{1\mathrm{DHM}}P_{2\mathrm{DHM}}}$ and $\bar{P_{1\mathrm{SEM}}P_{2\mathrm{SEM}}}$ are evaluated starting from the distances between their matrix indices. The magnification factor M_f_. is given by the ratio between these two distances. A number of elements have to be added, in order to magnify the less magnified image. The values of these added points are calculated using the nearest neighbor interpolation.

With this approach, a hypermatrix is obtained, where any (*i*,*j*) = (*x*,*y*) element corresponds both to z information and a gray scale. Finally, the texture of the phase map retrieved by DHM with the SEM image was implemented. Texture mapping is a technique for mapping a 2D image onto a 3D surface by transforming color data (in this case, the gray scale) so that it conforms to the surface plot.

## 4. Conclusions

The modeling of natural nanostructures with high performances, such as diatoms, butterflies and beetles, is indispensable for studying their photonic and mechanical properties. On the other hand, the design and fabrication of micro-opto-electro-mechanical systems (MOEMS), by either standard micromachining techniques or innovative ones such as 3D printing, requires the generation of 3D models with very detailed and highly resolved geometrical features. This step is unavoidable and necessary for the consequent fabrication processes. The proposed combination of SEM and DHM or AFM characterization techniques allows obtaining a 3D imaging of microscopic objects with xy SEM resolution (up to a few nanometers) and λ/20 resolution (typical of DHM) along the z direction. After the first characterization of the object with the two techniques applied separately, the two images are merged, making use of an original algorithm implemented with a commercially available software (i.e., MATLAB^®^). As examples, we presented the developed methodology and the combined final reconstruction applied on elements of biological interest, i.e., marine diatom valves belonging to three different species.
